# Electrochemical Regulation of Budding Yeast Polarity

**DOI:** 10.1371/journal.pbio.1002029

**Published:** 2014-12-30

**Authors:** Armin Haupt, Alexis Campetelli, Daria Bonazzi, Matthieu Piel, Fred Chang, Nicolas Minc

**Affiliations:** 1 Institut Jacques Monod, UMR7592 CNRS, Paris, France; 2 Institut Curie, UMR 144 CNRS/IC, Paris, France; 3 Department of Microbiology and Immunology, Columbia University College of Physicians and Surgeons, New York, New York, United States of America; Princeton University, United States of America

## Abstract

Cells are naturally surrounded by organized electrical signals in the form of local ion fluxes, membrane potential, and electric fields (EFs) at their surface. Although the contribution of electrochemical elements to cell polarity and migration is beginning to be appreciated, underlying mechanisms are not known. Here we show that an exogenous EF can orient cell polarization in budding yeast (*Saccharomyces cerevisiae*) cells, directing the growth of mating projections towards sites of hyperpolarized membrane potential, while directing bud emergence in the opposite direction, towards sites of depolarized potential. Using an optogenetic approach, we demonstrate that a local change in membrane potential triggered by light is sufficient to direct cell polarization. Screens for mutants with altered EF responses identify genes involved in transducing electrochemical signals to the polarity machinery. Membrane potential, which is regulated by the potassium transporter Trk1p, is required for polarity orientation during mating and EF response. Membrane potential may regulate membrane charges through negatively charged phosphatidylserines (PSs), which act to position the Cdc42p-based polarity machinery. These studies thus define an electrochemical pathway that directs the orientation of cell polarization.

## Abstract 2

Manipulation of yeast cell polarity by external electric fields reveals electrochemical pathways that influence the distribution of membrane lipids and the polarity regulator Cdc4p.

## Author Summary

The ability of cells to orient towards spatial cues is critical for processes such as migration, wound healing, and development. Although the role of electrochemical signals is well characterized in processes such as neuronal signaling, their function in cell polarity is much less understood or appreciated. Application of exogenous electric fields can direct cell polarization in many cell types, and electric fields of similar magnitude surround cells and tissues naturally. However, the significance and mechanism of these responses remain poorly understood. Here, we introduce budding yeast (*Saccharomyces cerevisiae*) as a powerful model system to study electrochemical regulation of cell polarity. We show that application of electric fields causes budding yeast to polarize in particular directions. We begin to identify key proteins involved in this response, which implicate an electrochemical pathway involving membrane potential, membrane charge, and an ion channel, which ultimately regulate the central polarity factor Cdc42p. These key proteins are not only needed for response to electric fields, but also contribute to cell polarity more generally. To test whether a change in membrane potential is sufficient to control cell polarization, we introduce a light-sensitive ion channel into yeast and show that we can now control the site of polarization simply by using a focused laser beam. Thus, our study shows that electrochemical regulation is an integral component of cell polarity pathways.

## Introduction

Cell polarization arises from the asymmetric accumulation of cellular components near a region of the plasma membrane. Although the roles of polarity proteins such as small GTPases and cytoskeletal elements have been studied extensively [1], much less is known about the possible contribution of electrochemical elements. Recent studies identifying certain ion transporters in regulating processes such as cell migration and polarized cell growth indicate potential roles of local pH, ion fluxes, and membrane potentials at the plasma membrane [2]–[8]. How these elements interface with established modules of polarity networks remains to be defined.

The importance of electricity in cell polarization is illustrated by the ability of electric fields (EFs) to direct cell polarization. It has been appreciated for decades that most cells—ranging from bacteria, fungi, and amoebas to animal cells—are electrotactic, and robustly orient polarity, migration, or division to applied exogenous EFs [9]–[14]. EFs of similar intensities as those used in these experiments naturally surround cells in tissues, and even individual cells such as fungal cells [10],[15],[16]. The physiological relevance of endogenous EFs has been demonstrated in fungal infection [17], immune cell response [18], wound healing, regeneration, and development [6],[10],[19],[20]. These findings have led to the proposal that in addition to responding to chemical and mechanical signals, cells may also be responding to endogenous electrotactic signals to guide cell polarization [20]. The response of cells to exogenous EFs provides a powerful tool to study electrochemical elements in cell polarization.

The molecular mechanisms of cell polarity are currently best understood in the budding yeast, *Saccharomyces cerevisiae*. Polarized cell growth in these cells is tightly controlled by intrinsic and extrinsic spatial cues. Haploid budding yeast cells display an axial budding pattern, in which new buds form adjacent to previous bud sites, while diploid cells exhibit a bipolar pattern, in which buds emerge at sites of previous division or growth [21],[22]. During mating, cells of opposite mating type polarize towards each other in response to gradients of secreted pheromones; exogenous application of the pheromone α-factor causes cells to grow a mating projection, forming a pear-shaped “shmoo.” The core polarity machinery required for both bud and shmoo formation is organized around the small GTPase Cdc42p, which coordinates actin assembly and exocytosis [23]–[25]. Bud site selection is specified by a Ras-like protein Rsr1p and its regulators [23]. During mating, these spatial cues used to direct budding are turned off, so that cells can polarize towards the mating partner. This reorientation of polarity involves Far1p and its interactions with the receptor-coupled Gβ protein and Cdc42 GEF [25]–[27]. As demonstrated by mutants affected in the regulation of only shmoos or only budding [23],[28],[29], there are specific molecular differences in the mechanisms governing budding and shmoo polarity. In general, still little is appreciated about electrochemical aspects of cell polarization in this cell type.

Here, we show that cell polarity can be directed by exogenous EFs in budding yeast. Although EFs have been shown to direct polarized growth in *Schizosaccharomyces pombe* [13] and *Candida albicans* [30],[31], there have been no reports to date in *S. cerevisiae*. We find that although EFs do not appear to affect wild-type (WT) budding cells, they do have robust effects on cells in the presence of pheromone and on mutants defective in bud site selection. We find a potassium channel and membrane lipid charges as components mediating EF responses. We further show, using a light-activated rhodopsin, that local membrane potential itself is capable of directing polarization. Our results demonstrate the importance of electrochemical signaling in cell polarity and begin to define mechanistically how they contribute to polarized cell growth.

## Results

### Electrotactic Responses of Budding Yeast Polarity

**Figure 1:**
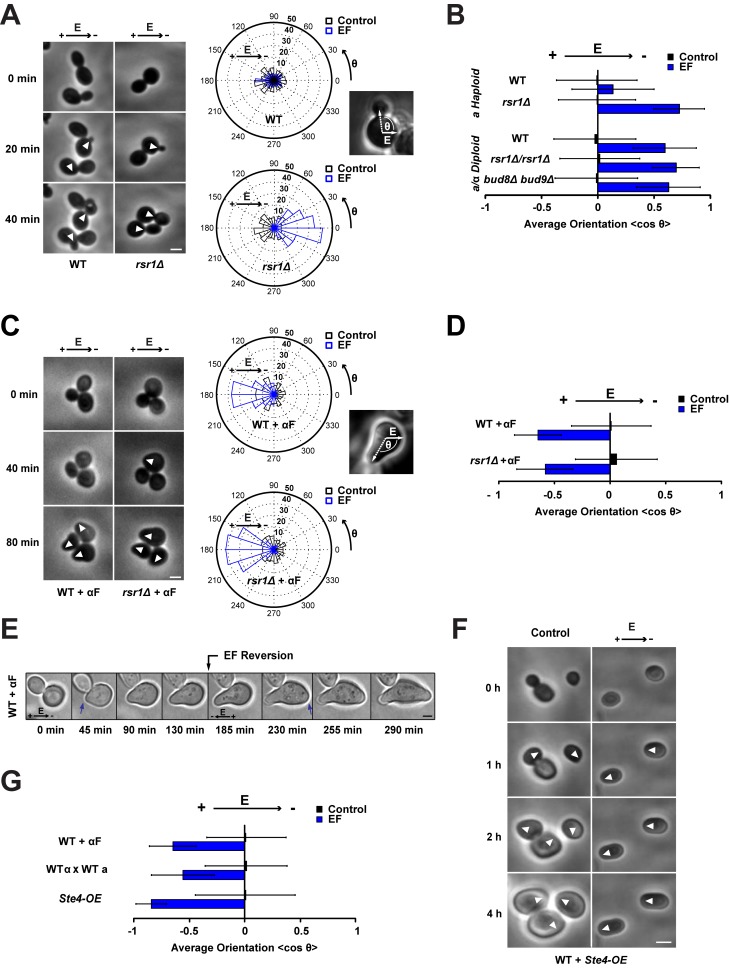
Budding versus shmooing yeast cells polarize in opposite directions in an electric field. (A) Phase contrast time lapse of WT and *rsr1*Δ budding yeast cells growing under an EF of 50 V/cm. White arrowheads point at sites of bud emergence. On the right are radial histograms of polarized growth direction (indicated as the final angle of bud emergence with the EF, θ) for WT and *rsr1*Δ cells in the presence or in the absence of an EF. (B) Average bud orientation, computed as <cosθ> after 3 h of growth in the absence or in the presence of an EF, for a population of haploids and diploids of the indicated genotype. A positive average orientation represents an orientation to the cathode (negative electrode of the EF), whereas a negative orientation stands for an orientation to the anode. (C) Phase contrast time lapse of WT and *rsr1*Δ budding yeast cells growing mating projections (“shmoos”) in the presence of α-factor (αF) under an EF of 50 V/cm. White arrowheads point at sites of shmoo emergence. On the right are radial histograms of polarized growth direction. (D) Average shmoo orientation after 3 h of mating tip growth in the absence or in the presence of an EF for a population of WT and *rsr1*Δ cells treated with α-factor. (E) Time-lapse images of shmoo reorientation in a WT cell after inverting the EF direction. A second shmoo is formed at the new anodal side after reversing the EF. Blue arrows indicate sites of shmoo emergence. (F) Time lapse of WT cells overexpressing Ste4p (Ste4-OE) in the absence or in the presence of an EF of 50 V/cm. White arrowheads point at sites of polarized growth. (G) Average shmoo orientation after 3 h in the absence or in the presence of an EF for a population of WT cells treated with α-factor, WT mating pairs, and WT cells overexpressing Ste4p. *n*>50 cells for all conditions. Error bars represent standard deviations. Scale bars: 2 µm.

We tested whether exogenous EFs can influence cell polarization in budding yeast. Yeast cells were grown in the presence of EFs in microfluidic channels, which allow for defined EF lines and heat control [13]. Haploid WT cells were mostly resistant to EF effects and budded at their normal axial position (Figure 1A and 1B). The bud site selection mutant *rsr1*Δ forms buds in random directions, in the absence of EF. In the EF, however, almost all new buds emerged at the cathode-facing side of the *rsr1*Δ cells after 1 h of exposure to an EF of 50 V/cm (Figure 1A and 1B; Movie S1). Cells did not exhibit any major signs of stress, cell death, or stress pathway activation [32], but grew with slightly reduced growth rates and prolonged cell cycle length as controls (Figure S1). Cathodal bud orientation displayed dose dependence on EF intensity and duration of application (Figure S2A and S2C). Diploid WT cells also polarized towards the cathode significantly more than WT haploids; this may reflect a less stringent regulation of budding pattern in diploids (Figure 1B). Thus, the EF was not able to efficiently override the normal spatial cues involved in axial budding, but could direct bud site polarization if these cues were absent or weak.

The application of EFs also directed the site of shmoo tip formation but, surprisingly, in the opposite direction. In the presence of uniform concentrations of α-factor and an EF, budding yeast cells showed a strong polarization towards the anode (Figures 1C, 1D, Figure S1S2B, S2D, and S2E; Movie S2). Changing the EF direction induced the formation of a second shmoo tip towards the new anode (Figure 1E; Movie S3). To rule out possible effects of adding mating factor exogenously, we also noted similar effects in mating pairs of cells. The EF disrupted mating and caused cells to polarize towards the anode of the EF instead of towards each other (Figures 1G and S2F). Cells that were induced to shmoo without external pheromones, by overexpressing Ste4p, the β subunit of the G protein involved in pheromone response, also polarized toward the anode [33] (Figure 1F and 1G; Movies S4 and S5). Thus, although bud and shmoo formation use many of the same components of the polarity machinery [21],[22], there is a striking difference in directionality (cathodal versus anodal) for how budding and shmooing yeasts respond to EFs.

### EF Response Involves the Cdc42p-Based Polarity Machinery

**Figure 2:**
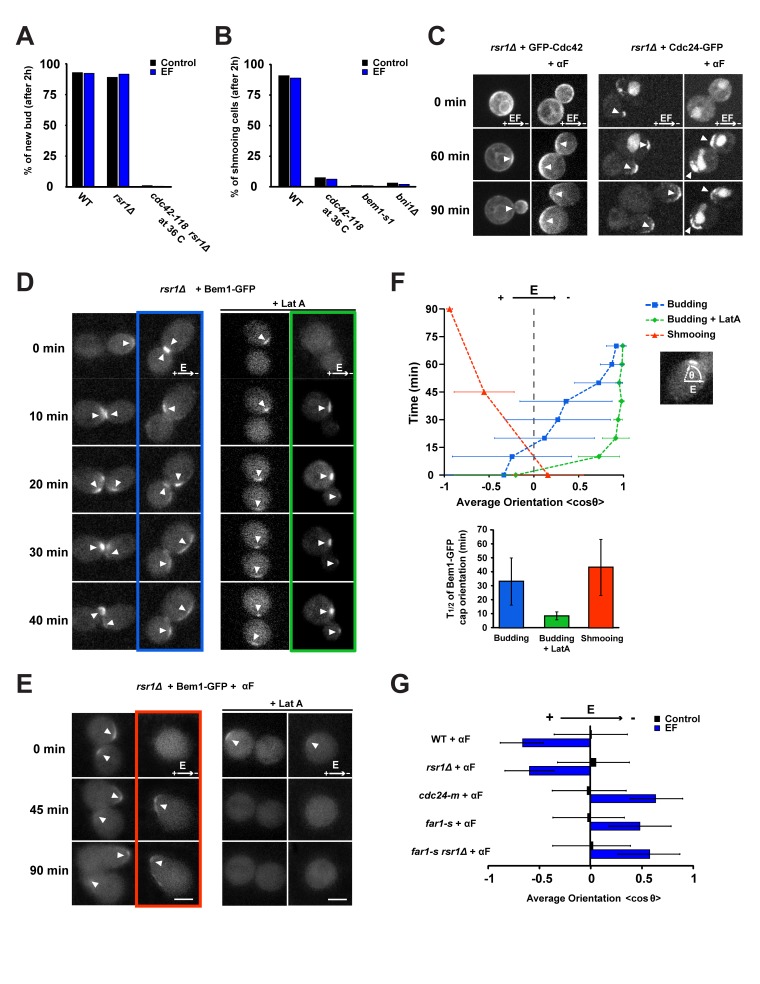
EF response involves Cdc42p polarization. (A) Percentage of new bud formation after 2 h in the absence or in the presence of an EF for a population of WT, *rsr1*Δ, and *cdc42-118 rsr1*Δ (at restrictive temperature, 36°C). (B) Percentage of shmoo formation after 2 h in the absence or in the presence of an EF for a population of WT, *cdc42-118* (at restrictive temperature), *bem1-s1*, and *bni1*Δ cells treated with α-factor (αF). (C) Confocal single plane time-lapse images of GFP-Cdc42 and Cdc24-GFP expressed in *rsr1*Δ cells grown under an EF, in the absence or in the presence of α-factor. White arrowheads indicate the successive positions of the protein polar caps. (D) Confocal single plane time-lapse images of Bem1-GFP in control and LatA-treated *rsr1*Δ cells grown in the absence and in the presence of an EF. White arrowheads indicate the successive positions of Bem1-GFP polar caps. (E) Confocal single plane time-lapse images of Bem1-GFP in control and LatA-treated *rsr1*Δ cells grown with or without an EF in the presence of α-factor. Note that LatA treatment induces rapid dispersion of the Bem1-GFP signal at the cap, with or without EF. White arrowheads indicate the successive positions of Bem1-GFP polar caps. (F) Temporal evolution of the average orientation of Bem1-GFP caps with respect to the applied EF in a population of *rsr1*Δ cells, treated with and without LatA or α-factor (top) (*n* = 13 cells for budding [blue], *n* = 9 cells for budding + LatA [green], *n* = 4 cells for shmooing [red]). Half-time (*t*_1/2_) corresponding to the mean orientation of Bem1-GFP polar caps to the cathode or anode of the EF is shown at the bottom. (G) Average shmoo orientation after 3 h in the absence or in the presence of an EF for a population of WT, *rsr1*Δ, *cdc24-m*, *far1-s*, and *rsr1*Δ *far1-s* cells treated with α-factor. *n*>50 cells for each condition. Error bars represent standard deviations. Scale bars: 2 µm.

We next tested whether cell polarization in response to EF requires the same polarity machinery normally used in budding or shmooing. The highly conserved small GTPase Cdc42p was required to polarize buds and shmoos in the absence or presence of the EF, as assessed with the loss-of-function mutant allele *cdc42-118* (Figures 2A, 2B, and S3A) [34]. In addition, mutants specifically defective in establishing polarity during mating but not budding, such as *bem1-s1* (a point mutant in the scaffold protein Bem1p [29]) and the formin null mutant *bni1*Δ [28], showed similar polarization defects in the absence or presence of the EF (Figures 2B, S3B, and S3E). Imaging GFP-Cdc42 [35] and the associated components Cdc24-GFP [36] (a GEF for Cdc42p) and Bem1-GFP [37] revealed that polarity caps assembled and oriented to the EF prior to bud or shmoo emergence (Figure 2C–2E). Bem1-GFP cap assembly was dependent on Cdc42p in the presence or absence of EF (Figure S3C and S3D). Actin also appeared to similarly mediate cell polarization in both instances. In budding cells, actin was dispensable for EF-induced Bem1-GFP cap cathodal orientation, although actin depolymerization appeared to accelerate polar cap accumulation at the cathodal side. In shmooing cells, actin inhibition caused rapid disappearance of the cap in the presence or absence of the EF [27] (Figures 2D, 2F, and S3F). Together, these data show that the EF acts in reorienting polarized cell growth through the normal polarity machinery, including Cdc42p and its regulators.

To investigate how EF directs mating projections, we tested the role of Far1p and Cdc24p. Mutant *far1-s* and *cdc24-m* cells have a specific orientation defect in response to α-factor, as they are not able to orient appropriately towards gradients of α-factor, and polarize instead using bud site selection cues [25]–[27]. In saturating concentrations of α-factor, we found that both of these mutants polarized towards the cathode of the EF (the opposite direction as WT cells) (Figure 2G). This reversal was also observed at non-saturating concentrations of pheromones (Figure S3G). As *rsr1Δ* mutants in the absence of α-factor bud towards the cathode, this suggests that *far1-s* and *cdc24-m* mutants may use machinery that orients buds to direct shmoo projections to the cathode.

### EF Response Involves the Membrane-Potential-Regulating Potassium Transporter Trk1p

**Figure 3:**
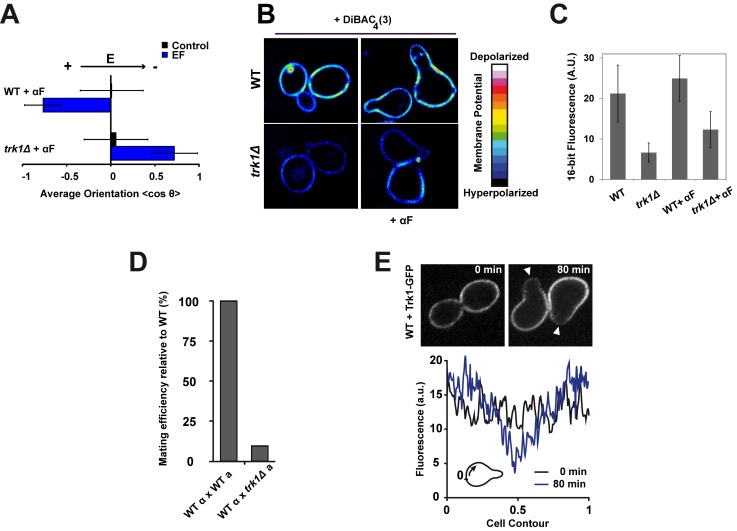
A potassium transporter, Trk1p, mediates EF response in shmoos. (A) Average shmoo orientation after 3 h in the absence or in the presence of an EF for a population of WT and *trk1*Δ cells treated with α-factor (αF) (*n*>50 cells). (B) Sixteen-color images of WT and *trk1*Δ cells stained with the membrane-potential-sensitive dye DiBAC_4_(3), which depicts reduced membrane fluorescence upon membrane hyperpolarization. (C) Quantification of DiBAC_4_(3) dye membrane staining intensity in WT and *trk1*Δ cells. (D) Mating efficiency of *trk1*Δ cells relative to WT. (E) Confocal single focal plane time-lapse images of Trk1-GFP in WT cells grown in the presence of α-factor. White arrowheads indicate shmoo growth sites. Below is the mean fluorescence intensity along the cell contour at times 0 and 80 min after α-factor treatment, averaged on five independent cells. Distances are normalized between 0 and 1 so that the value 0.5 corresponds to the site of shmoo emergence.

EFs are thought to affect cellular processes at or outside the plasma membrane, but not in the cell interior. They have been postulated to generate subcellular asymmetries in transmembrane potentials (TMPs) [13],[38],[39], and/or displace charged membrane proteins at the cell surface [40],[41]. To test whether membrane transporters mediate EF responses, we screened a set of well-characterized mutants and inhibitors affecting transport at the membrane. We found that calcium, sodium, and proton transport systems are not critical for EF sensing for bud or shmoo reorientation (Figure S4). We found, however, that a potassium transporter mutant *trk1*Δ was defective in the anodal orientation of mating projection, but not in budding orientation; these cells oriented shmoos to the cathode, in a similar manner as *far1-s* and *cdc24-m* mutants (Figure 3A; Movie S6). Trk1p is a high-affinity inward potassium transporter that displays conserved features in bacteria, plants, and fungi. In yeast, Trk1p is a major TMP regulator [42],[43], and *trk1*Δ cells exhibit hyperpolarized resting potential (Figure 3B and 3C) [42]. A *trk2*Δ mutant, in the secondary K^+^-import system (Trk2p), did not display any orientation defect in the EF, however [44]. Similarly to *far1-s* and *cdc24-m* mutants, *trk1*Δ mutants formed shmoos with normal morphology and timing, but were defective in mating (efficiencies of ∼10% of WT; Figure 3D), and displayed significant defects in polarizing in the correct direction in mating pairs (Figure S5B). In contrast, *trk1*Δ had no defects in bud emergence and haploid axial patterns (Figure S5A). We found that Trk1-GFP was located throughout the plasma membrane, but was reduced in emergent growing buds and shmoo tips, in a pattern similar to that of other membrane transporters [45],[46]. In shmooing cells, measurements of fluorescence intensity showed a stable back-to-front gradient, with a concentration ratio of about 3-fold (Figures 3E and S5C). In the presence of the EF, we observed a similar depletion of Trk1-GFP at the shmoo tip growing towards the anode, without noticeable change in protein distribution prior to tip growth (Figure S5D and S5E). Together, these data suggest that a natural gradient of Trk1p leading to local differences in potassium import may contribute to polarity regulation for shmoo tip orientation and EF response.

To shed more light on why cells may polarize in these different directions, we performed computational simulations and analytical calculations of the local EF strengths and electric potentials along the membrane of *S. cerevisiae* cells (Figure S6). This showed that sites of bud and shmoo emergence correspond to the minimum and maximum local EF potentials, and to sites of depolarized and hyperpolarized TMPs, respectively. This analysis thus led to the prediction that if EF-induced polarity is sensitive to TMPs, shmoos should emerge at sites of hyperpolarized TMP, while buds should emerge at sites of depolarized TMP.

### Asymmetries in Membrane Potential Can Direct Polarity

**Figure 4:**
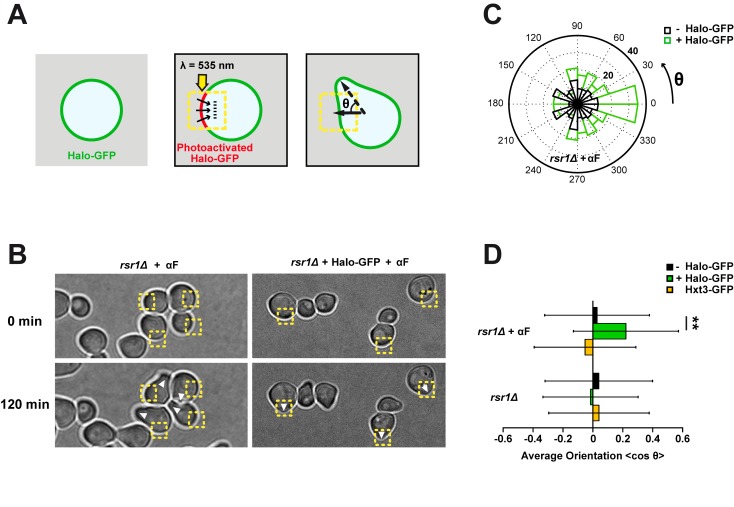
An optogenetic assay shows that asymmetries in membrane potential can direct polarity. (A) Optogenetic assay to generate asymmetries in membrane potential and assess for effect on polarity. Schematic representation of the experimental setup: a yellow laser (λ = 535 nm) is used to photoactivate Halorhodopsin (Halo) in selected regions of *rsr1*Δ cells. θ is the final angle of shmoo or bud emergence with respect to the direction of the photoactivated region. (B) *rsr1*Δ (left) and Halorhodopsin-GFP-expressing *rsr1*Δ (right) cells in the presence of α-factor (αF) and retinal are continuously photoactivated from time 0 to 20 min at the indicated yellow region. After 2 h, shmoos grow and polarity orientation can be quantified with respect to the photoactivated region. White arrowheads indicate sites of shmoo formation. (C) Quantification of optogenetic experiments: radial histogram of polarized growth orientation with respect to photoactivation angle in *rsr1*Δ and *rsr1*Δ + Halorhodopsin-GFP cells treated with α-factor. (D) Average orientation of polarized growth in budding and shmooing cells after 2 h of growth following local photoactivation for a population of *rsr1*Δ, *rsr1*Δ Hxt3-GFP, and *rsr1*Δ + Halorhodopsin-GFP cells (*n*>70 cells gathered from four independent datasets for all conditions and *n* = 166 cells gathered from seven independent experiments for *rsr1*Δ + Halorhodopsin-GFP + α-factor). **Student's *t* test, *p*<0.05. Error bars represent standard deviations.

To directly test the nature of the electrochemical signaling orienting polarity, we developed an optogenetic approach to locally modulate TMPs and/or ion fluxes [47]. Microbial opsins are light-gated transmembrane channels or pumps that have been used to modulate TMPs for neuron activation or silencing [48], as well as in other cell types such as yeast [49],[50]. We expressed different opsins tagged with GFP, and found that Halorhodopsin-GFP (NpHR) displayed the most robust expression and plasma membrane targeting, although there was some low level accumulation of Halorhodopsin-GFP in internal membranes, as often seen in other cell types [51] (Figure S7A). Halorhodopsin is a reversible inward chloride pump that causes rapid hyperpolarization of the TMP upon activation with green/yellow light [48]. We confirmed that Halorhodopsin could drive membrane hyperpolarization upon light activation in budding yeast, by measuring changes in global membrane potential in single cells following laser exposure, using the sensitive dye DiBAC_4_(3) (Figure S7B and S7C). We implemented a photoactivation assay to locally hyperpolarize mating and budding yeast cells at specific sites on the plasma membrane [52]. Cells were illuminated on a small square-shaped region at the cell surface with a yellow laser for 20 min, and subsequently filmed for 2 h to compute polarized growth orientation (Figure 4A and 4B). Laser exposure did not cause the cells to die or halt growth, but we did note a reduction in growth rate of ∼10%–15% in cells exposed to the laser compared to non-exposed controls in the same field. Accordingly, measurement of stress pathway activation revealed a minor stress response that remained negligible compared to typical osmotic stress responses (Figure S8A–S8C).

Strikingly, many cells expressing Halorhodopsin subsequently grew mating projections towards the site of the laser illumination (Figure 4C and 4D). This effect on orientation caused by light was similar to the one caused by 20 min of EF exposure (Figure S2D). Control cells that either did not express Halorhodopsin or expressed an unrelated GFP-tagged transmembrane protein, Hxt3-GFP, with similar localization [53] polarized in directions independent of the laser, showing that this effect was opsin-dependent and not due to cellular damage from the laser itself [54] (Figures 4D and S8D). Similar treatments in budding cells did not orient bud site emergence however (Figure 4D). These data suggest that the direction of mating projections can be controlled by local hyperpolarization of membrane potentials.

### Membrane Potential May Influence Lipid-Mediated Membrane Surface Charge to Steer Cdc42p Polarity Caps

**Figure 5:**
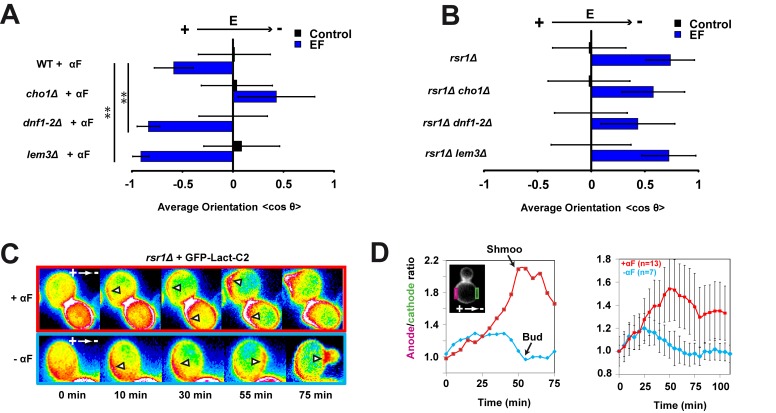
Membrane hyperpolarization orients polarity through local phosphatidylserine accumulation. (A) Average shmoo orientation in the absence or presence of an EF for a population of WT, *cho1*Δ, *dnf1-2*Δ, and *lem3*Δ cells treated with α-factor (αF) (*n*>50 cells). (B) Average bud orientation after 3 h in the absence and in the presence of an EF for a population of *rsr1*Δ, *rsr1*Δ *cho1*Δ, *rsr1*Δ *dnf1-2*Δ, and *rsr1*Δ *lem3*Δ cells (*n*>50 cells). (C) Sixteen-color epifluorescence time lapses of shmooing and budding cells polarizing in EFs and expressing GFP-Lact-C2 probe (a marker for PS). White arrowheads point at sites of PS accumulation. (D) Quantification of PS localization in EFs. The ratio of anodal versus cathodal signal is computed by measuring the total amount at the membrane on both facing sides of the cell. Left: ratio evolution for the depicted sequences in (C). The black arrows indicate the moment when shmoo tip or bud was first visible. Right: average ratio of anodal versus cathodal PS signal for shmooing and budding cells. **Student's *t* test, *p*<0.001. Error bars represent standard deviations.

We next asked how local changes in membrane potential influence the Cdc42-based polarity machinery. Although membrane potential could impact proton transport and local pH [13] or the transport of other ions, our candidate screen did not reveal any obvious role for proton or other ion transport systems other than Trk1p (Figure S4C and S4D). Another way by which membrane potential may affect polarity is through membrane electrostatics by affecting charged lipid flipping [55]–[57]. PS is a negatively charged lipid that acts as an electrostatic platform at the inner leaflet to regulate membrane binding of proteins including Cdc42p [58]. In budding yeast, PS concentrates at sites of shmoo and bud emergence [58]. A PS synthesis mutant *cho1*Δ has defects in Cdc42p recruitment, shmoo polarity, and mating [58]. We found that this mutant also exhibited an abnormal EF response in that it oriented mating projections to the cathode of the EF, much like *trk1*Δ, *far1-s*, and *cdc24-m* mutants (Figure 5A). Conversely, mutants in a lipid flippase complex, *dnf1-2*Δ or *lem3*Δ, which may have increased PS and negative surface charges [59], showed significant increased anodal shmoo orientation in the EF. PS and membrane charges affected EF response only in shmoos, not in buds (Figure 5B). Next, we imaged PS localization using a GFP-Lact-C2 probe [58],[60]. In shmooing cells, PS rapidly accumulated and persisted at the anodal side, long before shmoo appearance. In budding cells, PS also initially accumulated at the anodal side, but then reverted to the cathodal side immediately prior to bud emergence, often leaving a secondary patch at the anodal side (Figure 5C and 5D). Thus, asymmetries in membrane potential may bias the localization of Cdc42p and other polarity factors through effects on PS and membrane charge.

## Discussion

**Figure 6:**
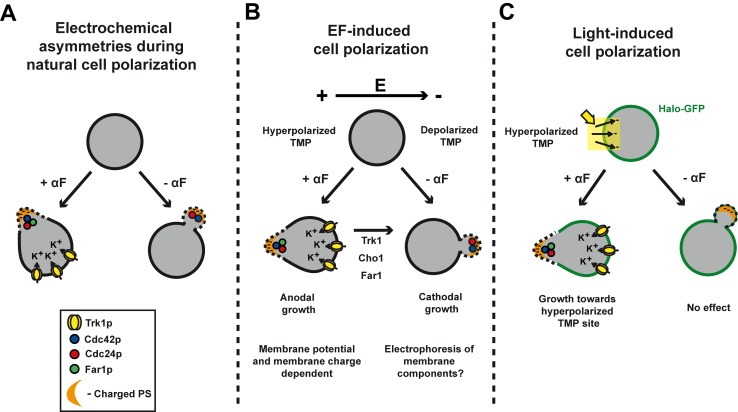
Influence of electrochemical asymmetries on polarity. (A) During normal cell polarization, electrochemical layers segregate to the front and the back of the cell and may influence polarization processes, for instance during mating. (B) In an EF, the anode-facing side has hyperpolarized membrane potential, which drives anodal growth of the shmoos, in a Trk1-, Cho1*-*, and Far1-dependent manner. The secondary default orientation mode appears to be the cathodal orientation, which drives bud emergence and shmoo growth in *trk1*Δ, *cho1*Δ, and *far1-s* mutants by a yet unknown mechanism. (C) Optogenetic experiments directly suggest that local hyperpolarization of cell membrane potential can drive shmoo polarized growth but not bud site emergence. αF, α-factor.

These are the first studies, to our knowledge, showing the input of membrane electrochemistry in the regulation of cell polarity in *S. cerevisiae*. We find that EFs direct the site of bud formation and mating projections in different directions. Our optogenetic experiments further show that in shmooing cells, local hyperpolarization of membrane potential is actually sufficient for polarity reorientation (Figure 6). The mating defects of *trk1*Δ and *cho1*Δ mutants [58], for instance, demonstrate that this pathway contributes to cell polarization even in the absence of EFs. Our results suggest a model in which the asymmetric segregation of Trk1p and possibly other transporters produces positive charges at the back of the cell and negative charges on PS lipids at the front of the cell, which promotes the polarized distribution of Cdc42. In the absence of EFs, the asymmetry of Trk1p localization may arise from initial polarization of membrane insertion. These electrochemical pathways may thus represent a positive feedback loop that stabilizes the axis of Cdc42-based polarity for chemotropism.

A surprising finding of this study is the different behavior of budding versus shmooing cells. Although these polarization systems share downstream polarity regulators, we found clear differences in the requirement for upstream electrochemical elements. *rsr1*Δ cells bud towards the cathode, while the same strain shmoos towards the anode. Mutations in Far1, Cdc24, Trk1, and Cho1 all cause cells to shmoo in an abnormal direction in response to EF and have mating defects in the absence of EF, but have little or no effect on bud site selection [27],[58]. The cathodal orientation of budding cells in EFs suggests that buds originate at sites of depolarized TMP. However, genetic and optogenetic analysis demonstrate that they may not be dependent on gradients of membrane potential or PS levels, suggesting that regulation of bud site selection is determined by a distinct mechanism. Although it is not yet known what elements act upstream of Cdc42p to drive cathodal growth, a plausible hypothesis is that EFs may localize some charged membrane proteins by direct electrophoresis, as suggested in other systems [13],[40]. The EF thus causes a tug-of-war between two competitive pathways that steer polarity in different directions, with the anodal one being dominant in response to mating factors.

The observed responses of cells to exogenously applied EFs lead to a question of whether EFs normally contribute to polarity regulation. Tissues and even individual polarized cells are surrounded by EFs, which may arise from asymmetries in ion transport [61]–[65]. We speculate that fungi may respond to their own EFs, possibly during mating, in the context of fungal communities such as biofilms, and in their natural environment to guide them during invasion of host tissues, for instance. It would be interesting to examine the role of genes such as Trk1 on various fungal behaviors.

Mechanisms of electrochemical regulation of cell polarity are likely conserved. Our data are consistent with recent findings implicating a similar set of actors in fission yeast, neutrophils, keratocytes, and slime molds [9],[10],[12],[13],[40]. Fission yeasts respond to EFs by orienting their growth axis perpendicular to the EF, producing bent morphologies. Cdc42, formins, and the Pma1 proton pump at the plasma membrane are identified as critical elements. Pma1 affects cell polarity and actin assembly even in the absence of exogenous EFs, indicating a role for membrane potential and intracellular pH in regulating normal tip growth [13]. Migrating neutrophils, keratocytes, and slime molds orient migration to exogenous EFs, possibly through effects on membrane potential [10],[66]. These responses involve the phosphorylation and charge additions on phosphatidylinositol lipids—mediated by PI3-kinase—that recruit and activate Rho GTPases for polarized migration.

Recent studies on plasma membrane pumps and channels are beginning to reveal the pivotal role of membrane potential, pH, and/or local ion transport in cell migration [67]–[69], mitotic rounding [70], asymmetric aging [71], and tissue patterning [4],[7],[72]. A Na^+^-H^+^ exchanger, Nhe1, is needed for directionality in fibroblast migration; this transporter has been shown to control local pH, which affects the ability of a Cdc42 GEF to bind to the plasma membrane [69],[73]. Similarly, the membrane targeting of Dishevelled needed for planar cell polarity activation in fly epithelia may rely on charge interaction and pH [4],[69]. An inward-rectifier potassium channel influences patterning of zebrafish skin stripes, leading to a model in which membrane potential controls a directional switch in cell migration and consequent cell–cell arrangement in the tissue [7]. The establishment of a highly tractable system in yeast to study the mechanisms of electrochemical regulation will serve as a foundation to understand the diverse roles of membrane electrochemistry in processes related to cell polarity.

## Materials and Methods

### Yeast Strains, Media, and Genetic Methods

Standard methods for *S. cerevisiae* media and genetic manipulations were used. Strains and plasmids used in this study are listed in Tables S1 and S2, respectively.

### Microscopy

Microscopy was performed at room temperature (23–25°C) with either an inverted wide-field fluorescence microscope or a spinning-disk confocal microscope. Images were acquired, processed, and analyzed with Micro-Manager or Metamorph.

### Electric Field Chambers

Chambers to apply the EF to the cells were adapted from previously described methods [13]. Microchannels were approximately 200 µm high, 500 µm wide, and 4 cm long and were fabricated in PDMS. *S. cerevisiae* cells were immobilized by adding 1% of low-melting agarose to the medium. For shmooing experiments with saturating pheromones, 50 µM of α-factor was added to the medium, and cells were placed in the channel 30 min prior to EF application. Because of their slow growth, *cho1*Δ cells were placed in the channel 1 h prior to EF application. Reservoirs connecting electrodes to the channels contained 4% agarose blocks made of medium, which protect cells in the channel from potentially toxic products emanating from the electrodes. Electrodes connected to a generator were immersed in liquid medium added on top of the reservoirs. In these conditions, growth rate and cell cycle periods were almost unaffected, and no significant stress was induced (Figure S1).

### Optogenetics

The optogenetic assay used a 535-nm laser, with a power of ∼5 mW, interfaced with an iLas system (Roper Scientific) mounted on a confocal spinning disk and a 63× objective. This allowed irradiation of multiple regions of interest (of 20×20 px^2^) in a given field of view. Cells were placed on a 2% agar pad containing 20 µM of all-trans retinal and 50 µM of α-factor for shmooing experiments. Cells were put on the pad 30 min prior to laser excitation. The laser was turned on for a continuous period of 20 min, and the cells were subsequently filmed for 2 h to monitor polarized growth. Laser exposure did not induce major changes in growth rate or stress levels (Figure S8A–S8C).

### Pharmacological Inhibitors

All inhibitors were prepared at the indicated concentration and applied before EF application. Latrunculin A (LatA) (Sigma) was used at a final concentration of 100 µM from a 100× stock in DMSO. The calcium ionophore A23187 was used at a final concentration of 10 µM. The calcium chelating agent EGTA was used at a final concentration of 2 mM.<1?tpb +2pt?>

### Quantitative Mating Assays

Efficiency of mating in *trk1*Δ mutants was assayed by quantitative counting of mating diploids. WT Mat α (AC 131), WT Mat a (AC 129), and *trk1*Δ Mat a (AC 31) cells were grown to mid-log phase in YPD medium and concentrated to 10 OD/ml; WT Mat α cells were incubated at a 10∶1 ratio with target WT or *trk1*Δ Mat a cells, and collected into a soft pellet by centrifugation. After 4 h of mating at 30 °C, cells were suspended in liquid YPD, and serial dilutions were plated on medium selective for diploids. Mating efficiency was compared between WT and *trk1*Δ by counting the number of diploid colonies obtained at different dilutions. About 500 colonies were counted for each condition, and the assay was repeated twice.

In addition to this assay, we also counted the number of genuine zygotes by microscopy after 4 h of mating. To this aim, WT Mat α, WT Mat a, and *trk1*Δ Mat a cells were grown in YPD liquid medium to mid-log phase and concentrated to 10 OD/ml. WT Mat α cells were stained with calcofluor for 5 min, subsequently rinsed with YPD, and incubated at a 10∶1 ratio with target WT or *trk1*Δ Mat a cells. A 10-µl drop of each mixture was then spotted onto a YPD plate and incubated for 4 h at 30 °C. The mixtures were then imaged on a microscope, and mating efficiency was computed as the ratio of genuine zygotes to the total number of Mat α cells in the field of view. About 500 cells were counted for each condition, and the assay was repeated twice. This assay yielded a mating efficiency in the *trk1*Δ of about 30% of the WT.

### Budding Patterns

To test the role of Trk1p in axial budding, we generated a *trk1*Δ strain in the W303 background with a WT copy of the BUD4 gene (AC 134). Cells were then grown to mid-log phase in YPD medium and stained with calcofluor for 5 min to mark bud scars. Axially budding cells were counted when more than three scars were clustered at one site on the surface.

### Chemotropism Efficiency in Zygotes

To compare the efficiency of chemotropism in WT versus *trk1*Δ cells, we used a previously described assay that takes advantage of the fact that WT cells grow towards their mating partners irrespective of previous bud site selection, while mutants with defective mating polarity (like *far1-s* or *cdc24-m*) use bud site selection cues to grow shmoos [74]. WT Mat α, WT Mat a, and *trk1*Δ Mat a cells were grown in YPD liquid medium to mid-log phase and concentrated to 10 OD/ml. WT Mat a and *trk1*Δ Mat a cells were stained with calcofluor for 5 min, subsequently rinsed with YPD, and incubated at a 1∶1 ratio with target WT Mat α cells. A 10-µl drop of each mixture was then spotted onto a YPD plate and incubated for 4 h at 30°C. The mixtures were then imaged to assess the position of the bud scar relative to the fusion site in each newly formed zygote. Only zygotes with a single fluorescent bud scar were counted. Zygotes were scored as proximal if the bud scar was in the one-third of the cell adjacent to the fusion site, medial for the middle one-third, and distal for the one-third away from the fusion site (Figure S5B).

### Membrane Potential Measurement

To measure global membrane potential in single budding yeast cells, we used the membrane potential dye DiBAC_4_(3) (Invitrogen), which absorbs in blue light and depicts increased membrane fluorescence upon membrane depolarization, with a sensitivity of nearly 1% per millivolt [7]. Cells were incubated with a concentration of 50 µM dye for 30 min, and images were taken on a confocal spinning disk. Relative membrane potential values were then quantified as membrane signal subtracted from background signal. To assess membrane hyperpolarization by Halorhodopsin, cells were immobilized at the bottom of a microfluidic chamber, between a dialysis membrane and the coverslip [75]. Cells expressing Halorhodopsin-GFP were bleached by long-time exposure with a blue laser. Medium was subsequently exchanged with YPD containing 50 µM DiBAC_4_(3) dye, and the dye was left to stain the cells for 30 min. Dye staining intensity at the membrane was then measured in the same pre-bleached cells at two consecutive time points spaced by 3 min (*I*_0_ and *I*_1_) to compute dye photo-bleaching. These cells were then exposed to the yellow laser for 3 min, and the final dye staining was computed again (*I*_2_). The specific loss of dye staining associated with Halorhodopsin effects on membrane potential was then computed as 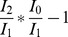, which is expected to be negative for membrane hyperpolarization and positive for membrane depolarization.

### Computer Simulations of EF Effects on Yeast Cells

Computer simulations were performed using the Matlab Partial Differential Equation Toolbox (MathWorks). The cell surfaces as well as the channel sides were considered as perfect insulators, while the cell interior and the surrounding medium as conductors.

### Analytical Calculations of EF Effects on Yeast Cells

The electric potential, Φ, created by the applied EF, 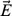, was analytically computed by solving the Laplace equation: ΔΦ  = 0, with the boundary condition at the insulating membrane 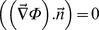, with 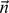 the vector normal to the membrane, and the limit condition at infinity 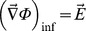. *S. cerevisiae* cells were represented by a sphere, leading to the classical results [76] for the potential at the membrane Φ_m_ and the field at the membrane 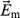: 
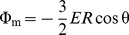
 and 
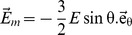
, with *R* the radius of the sphere and θ the angle with the field.

## Supporting Information

Figure S1 **EF effects on cell physiology.** (A) Effect of EFs on the timing of bud (dose-dependent, left) and shmoo emergence (100 V/cm, right). (B) Effect of EF (100 V/cm for 1 h) on stress levels of cells in presence of 50 µM α-factor as measured by Hog1-GFP nuclear accumulation. Osmotic stress (0.5 M NaCl for 5 min) is used as a positive control for stress. (C) Quantification of Hog1-GFP nuclear to cytoplasmic levels. *n*>25 cells for each condition. Error bars represent standard deviations.
(TIF)

Figure S2 **Polarity orientation to EFs displays dose dependence on EF strength and duration of application.** (A) Evolution of the average orientation of bud site emergence angles of *rsr1*Δ cells after 2 h under different EF strengths. (B) Evolution of the average orientation of shmoo tip growth angles of WT cells in the presence of α-factor after 2 h under different EF strengths. (C) Evolution of the average orientation of bud site emergence angles of *rsr1*Δ cells under an EF of 50 V/cm as a function of the duration of EF application. Orientation was measured 2 h after start of EF application. (D) Evolution of the average orientation of shmoo tip growth angles of WT cells in the presence of α-factor under an EF of 50 V/cm as a function of the duration of EF application. Orientation was measured 2 h after start of EF application. (E) Shmoo orientation of WT cells in EFs is independent of pheromone concentration. (F) Images of adjacent cells of opposite mating type in the absence or presence of EFs (left panel). Mat α cells were stained with calcofluor prior to the experiment. Note that control mating pairs polarize towards each other to form a zygote, while cells in EFs grow shmoo tips to the anode, and fail to mate. Right bar graph: percentage of mating cells after 3 h in no EF or an EF of 50 V/cm. *n*>50 cells for each condition. Error bars represent standard deviations. Scale bars: 2 µm.
(TIF)

Figure S3 **EF orients polarized growth through canonical downstream polarity effectors.** (A and B) Time lapses of indicated mutants grown in the absence or presence of exogenous EFs. Note that mutants that fail to polarize grow in a near isotropic manner, with no bud or shmoo tip emergence. (C and D) Time lapses of Bem1-GFP localization in the indicated mutants grown in the absence or presence of exogenous EFs at the restrictive temperature, 36°C. Note that Bem1-GFP fails to polarize in *cdc42-118rsr1*Δ cells independent of EF presence. White arrowheads indicate the successive positions of Bem1-GFP polar caps. Cells were cultured at 36°C for 1 h prior to EF assay. (E) Average orientation of bud site emergence angles in the indicated mutants (*n*>50 for each condition). (F) Control for the effect of LatA in the microfluidic set-up used for EF applications. Abp1 is a marker for actin patches that becomes diffuse when actin is fully depolymerized. (G) EF-dependent shmoo orientation of WT and *far1-s* cells is independent of pheromone concentration. Error bars represent standard deviations. Scale bars: 5 µm.
(TIF)

Figure S4 **Candidate screen for ion transport systems involved in EF response in buds versus shmoos.** (A and B) Strain background (W303 versus S2888c) does not impact EF orientation of buds or shmoos. (C) Average orientation of bud site emergence angles in the indicated mutants and drugs in an *rsr1*Δ background. (D) Average orientation of shmoo tip growth angles in the indicated mutants and drugs in the presence of α-factor in a WT background (*n*>50 for each condition). Drug concentrations are indicated in Materials and Methods. Error bars represent standard deviations.
(TIF)

Figure S5 **Effects of Trk1 on budding patterns and chemotropism during mating and dynamic localization of Trk1-GFP during bud and shmoo emergence.** (A) Axial budding pattern in *trk1*Δ cells expressing a WT BUD4 (strain AC 134). (B) Position of zygote fusion sites compared to previous bud scars in WT and *trk1*Δ cells. (C) Trk1-GFP signal is reduced at shmoo tips. Optical sections spaced by 200 nm were used for maximum intensity projections (left, “MAX”). The 19 individual sections are shown on the right. Three representative individual cells are depicted. (D) Changes of localization of Trk1-GFP during bud emergence in presence (in *rsr1*Δ background) or absence (WT background) of an EF. (E) Changes of localization of Trk1-GFP in shmooing cells exposed to an EF. Note the disappearance of Trk1-GFP at the shmoo tip growing to the anode.
(TIF)

Figure S6 **Computational simulation of EF effects on membrane potential predicts a hyperpolarization at the anode-facing side and a depolarization at the cathode-facing side.** (A) Computational simulation of the EF-induced electric potential (Φ) landscape around a *S. cerevisiae* cell created by an EF of 50 V/cm. The cytoplasm is set at an arbitrary homogenous reference potential. The lines represent the equipotentials. (B) Predicted local changes in extra-transmembrane potential created by the EF.
(TIF)

Figure S7
**Optimization of optogenetic control of membrane potential in** ***S. cerevisiae.*** (A) Images of WT cells expressing different opsins (Archaerhodopsin, Channelrhodopsin, and Halorhodopsin—from left to right) tagged with GFP, after 18 h of induction at 25°C. (B) Assay to monitor Halorhodopsin light-induced membrane depolarization in single cells using DiBAC_4_(3). Cells expressing Halorhodopsin-GFP are placed in a microfluidic flow chamber (see Materials and Methods), and the GFP signal is first bleached with 15 stacks of 5-s exposure with a blue laser. Cells are subsequently rinsed with DiBAC_4_(3) dye and left to stain for 30 min. Effects of dye photo-bleaching are accounted for by taking single slices spaced apart by 3 min, and measuring membrane intensity subtracted from background before and after the 3-min interval (*I*_0_ and *I*_1_). Hyperpolarization induced by yellow light activation of Halorhodopsin is then assessed by exposing cells to a yellow laser for 3 min, and measuring fluorescence in the green channel (*I*_2_). Specific loss of fluorescence associated with membrane hyperpolarization is computed as 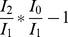, which accounts for dye photo-bleaching, and is expected to be positive upon membrane depolarization and negative upon membrane hyperpolarization. (C) Halorhodopsin activation triggers hyperpolarization of *rsr1*Δ cells. Fluorescence changes are computed as described in (B). *p*-Value is 0.079 as calculated by Student's *t* test. Error bars represent standard deviations, and *n*≥32 cells were analyzed.(TIF)

Figure S8 **Effects of optogenetic assays on cell physiology.** (A) Effect of locally restricted yellow light exposure for 20 min on the growth rate of shmoos. (B) Effect of local yellow light exposure (yellow boxes) for 20 min on stress levels of cells in presence of 50 µM α-factor as measured by Hog1-GFP nuclear accumulation. Osmotic stress (0.5 M NaCl for 5 min) is used as a positive control for stress. (C) Quantification of Hog1-GFP nuclear to cytoplasmic levels. (D) Subcellular localization of Hxt3-GFP expressed under its endogenous promoter in *rsr1*Δ cells. *n*>25 cells for each condition. Error bars represent standard deviations.
(TIF)

Table S1 **Strains used in this study.**
(XLSX)

Table S2 **Plasmids used in this study.**
(XLSX)

Movie S1 **Haploid** ***rsr1*** **Δ** ***S. cerevisiae*** **cells budding toward the cathode of the EF.** Elapsed time  = 180 min. Time is in hours: minutes.
(AVI)

Movie S2 **Haploid MAT a WT** ***S. cerevisiae*** **cells shmoo toward the anode of the EF in the presence of α-factor.** Elapsed time  = 160 min. Time is in hours: minutes.
(AVI)

Movie S3 **Inducing two sites of polarization by switching the direction of the EF.** Cell in α-factor and EF. After 140 min, the EF was reversed. Elapsed time  = 290 min. Time is in hours: minutes.
(AVI)

Movie S4 **WT cells overexpressing Ste4p fail to stabilize polarity at a single place and grow successive shmoos all around the surface.** Elapsed time  = 240 min. Time is in hours: minutes.
(AVI)

Movie S5 **WT cells overexpressing Ste4p and grown in the EF stabilize shmoo growth towards the anode.** Elapsed time  = 190 min. Time is in minutes.
(AVI)

Movie S6 **Merged movie depicting haploid MAT a WT cells growing shmoos toward the anode of the EF and subsequently haploid MAT a** ***trk1*** **Δ cells growing shmoos toward the cathode.** Time is in minutes.
(AVI)

Data S1 **Excel spreadsheet containing, in separate sheets, the underlying numerical data and statistical analysis for** **Figures 1B, 1D, 1G** **,** **2A, 2B, 2F, 2G** **,** **3A, 3C, 3D, 3E** **,** **4D** **,** **5A, 5B, 5D** **, **S1A, S1C**, **S2A, S2B, S2C, S2D, S2E, S2F**, **S3E, S3G**, **S4A, S4B, S4C, S4D**, **S5A, S5B**, **S7C**, **S8A, and S8C**.**
(XLSX)

## Abbreviations

EF: electric field
LatA: latrunculin A
PS: phosphatidylserine
TMP: transmembrane potential
WT: wild-type
