# Supplementary figures and images for: Electrochemical Regulation of Budding Yeast Polarity
Source: PLoS Biol. 2014 Dec 30;12(12):e1002029. doi: 10.1371/journal.pbio.1002029 (PMC4280105; doi:10.1371/journal.pbio.1002029)

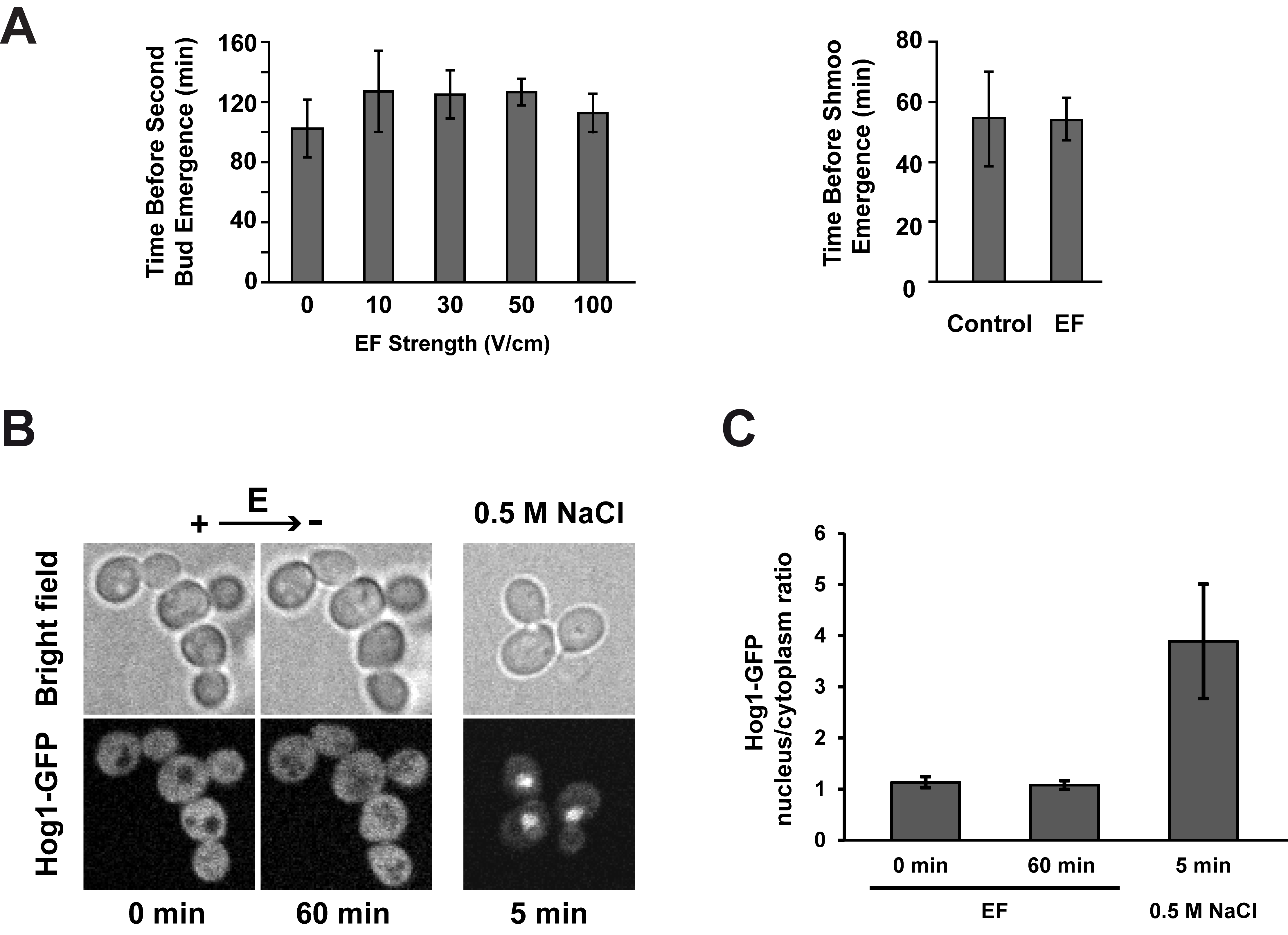

Supplement: Figure S1 — EF effects on cell physiology. (A) Effect of EFs on the timing of bud (dose-dependent, left) and shmoo emergence (100 V/cm, right). (B) Effect of EF (100 V/cm for 1 h) on stress levels of cells in presence of 50 µM α-factor as measured by Hog1-GFP nuclear accumulation. Osmotic stress (0.5 M NaCl for 5 min) is used as a positive control for stress. (C) Quantification of Hog1-GFP nuclear to cytoplasmic levels. n>25 cells for each condition. Error bars represent standard deviations. (TIF) [file pbio.1002029.s001.tif]

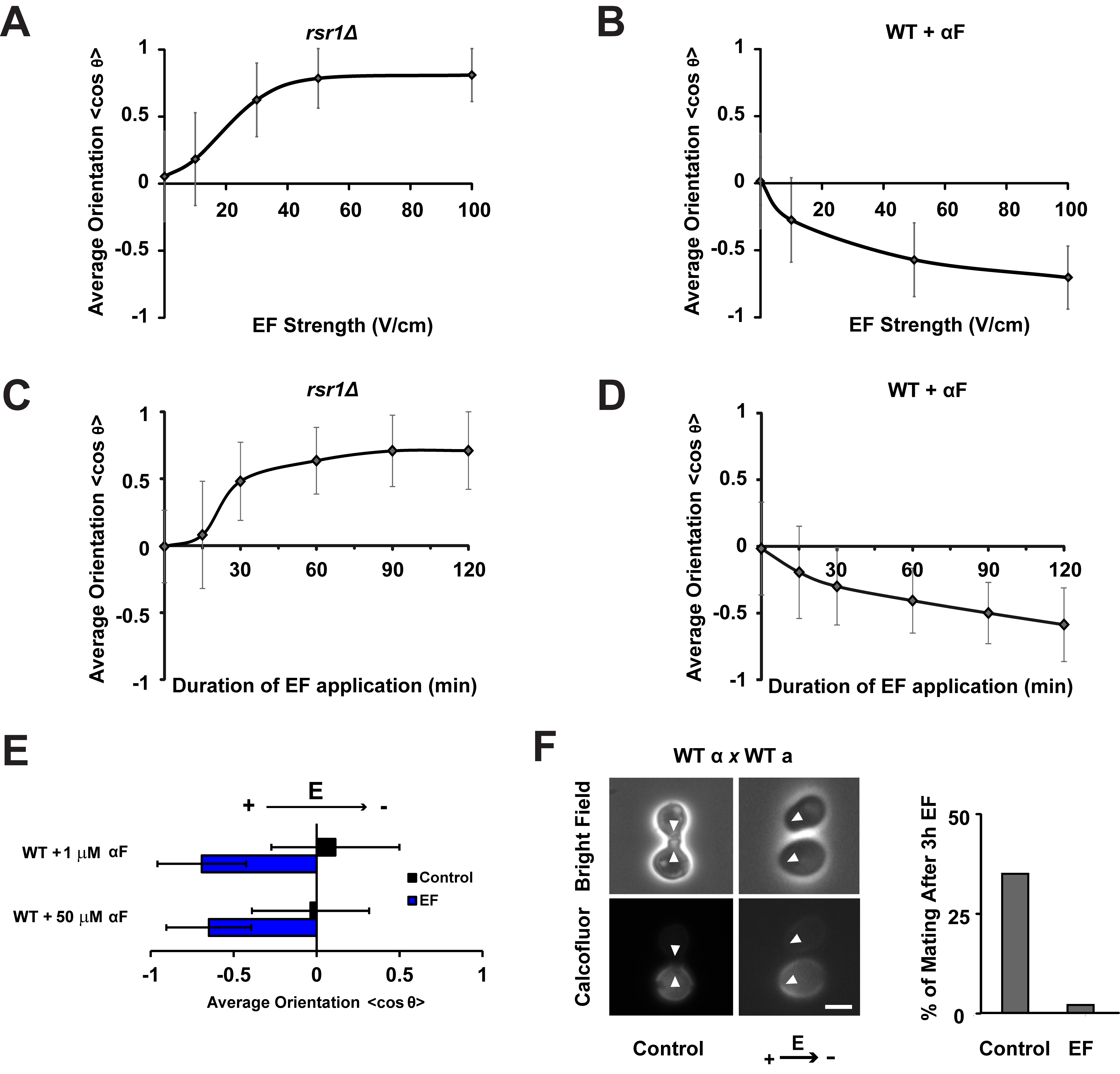

Supplement: Figure S2 — Polarity orientation to EFs displays dose dependence on EF strength and duration of application. (A) Evolution of the average orientation of bud site emergence angles of rsr1Δ cells after 2 h under different EF strengths. (B) Evolution of the average orientation of shmoo tip growth angles of WT cells in the presence of α-factor after 2 h under different EF strengths. (C) Evolution of the average orientation of bud site emergence angles of rsr1Δ cells under an EF of 50 V/cm as a function of the duration of EF application. Orientation was measured 2 h after start of EF application. (D) Evolution of the average orientation of shmoo tip growth angles of WT cells in the presence of α-factor under an EF of 50 V/cm as a function of the duration of EF application. Orientation was measured 2 h after start of EF application. (E) Shmoo orientation of WT cells in EFs is independent of pheromone concentration. (F) Images of adjacent cells of opposite mating type in the absence or presence of EFs (left panel). Mat α cells were stained with calcofluor prior to the experiment. Note that control mating pairs polarize towards each other to form a zygote, while cells in EFs grow shmoo tips to the anode, and fail to mate. Right bar graph: percentage of mating cells after 3 h in no EF or an EF of 50 V/cm. n>50 cells for each condition. Error bars represent standard deviations. Scale bars: 2 µm. (TIF) [file pbio.1002029.s002.tif]

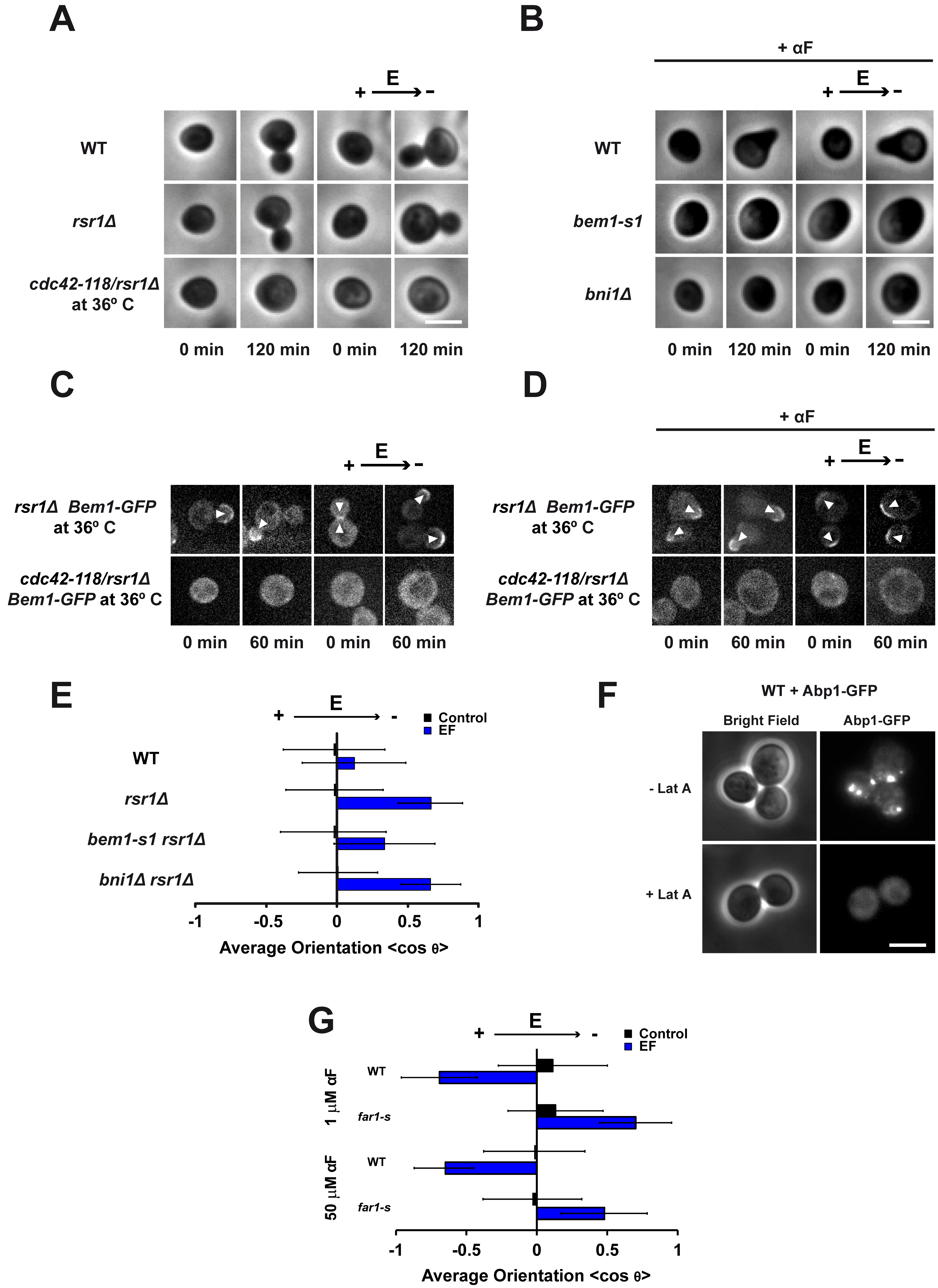

Supplement: Figure S3 — EF orients polarized growth through canonical downstream polarity effectors. (A and B) Time lapses of indicated mutants grown in the absence or presence of exogenous EFs. Note that mutants that fail to polarize grow in a near isotropic manner, with no bud or shmoo tip emergence. (C and D) Time lapses of Bem1-GFP localization in the indicated mutants grown in the absence or presence of exogenous EFs at the restrictive temperature, 36°C. Note that Bem1-GFP fails to polarize in cdc42-118rsr1Δ cells independent of EF presence. White arrowheads indicate the successive positions of Bem1-GFP polar caps. Cells were cultured at 36°C for 1 h prior to EF assay. (E) Average orientation of bud site emergence angles in the indicated mutants (n>50 for each condition). (F) Control for the effect of LatA in the microfluidic set-up used for EF applications. Abp1 is a marker for actin patches that becomes diffuse when actin is fully depolymerized. (G) EF-dependent shmoo orientation of WT and far1-s cells is independent of pheromone concentration. Error bars represent standard deviations. Scale bars: 5 µm. (TIF) [file pbio.1002029.s003.tif]

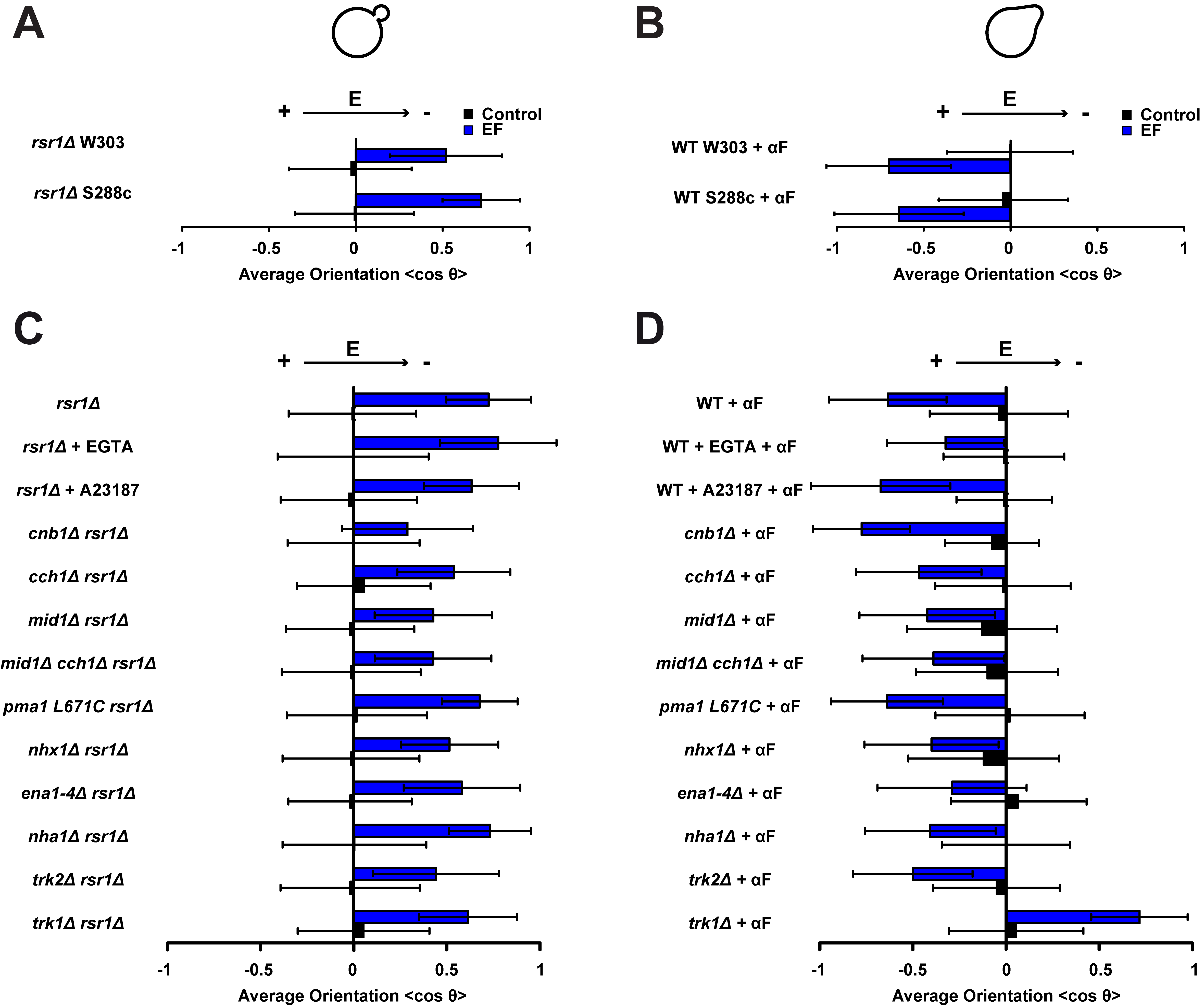

Supplement: Figure S4 — Candidate screen for ion transport systems involved in EF response in buds versus shmoos. (A and B) Strain background (W303 versus S2888c) does not impact EF orientation of buds or shmoos. (C) Average orientation of bud site emergence angles in the indicated mutants and drugs in an rsr1Δ background. (D) Average orientation of shmoo tip growth angles in the indicated mutants and drugs in the presence of α-factor in a WT background (n>50 for each condition). Drug concentrations are indicated in Materials and Methods. Error bars represent standard deviations. (TIF) [file pbio.1002029.s004.tif]

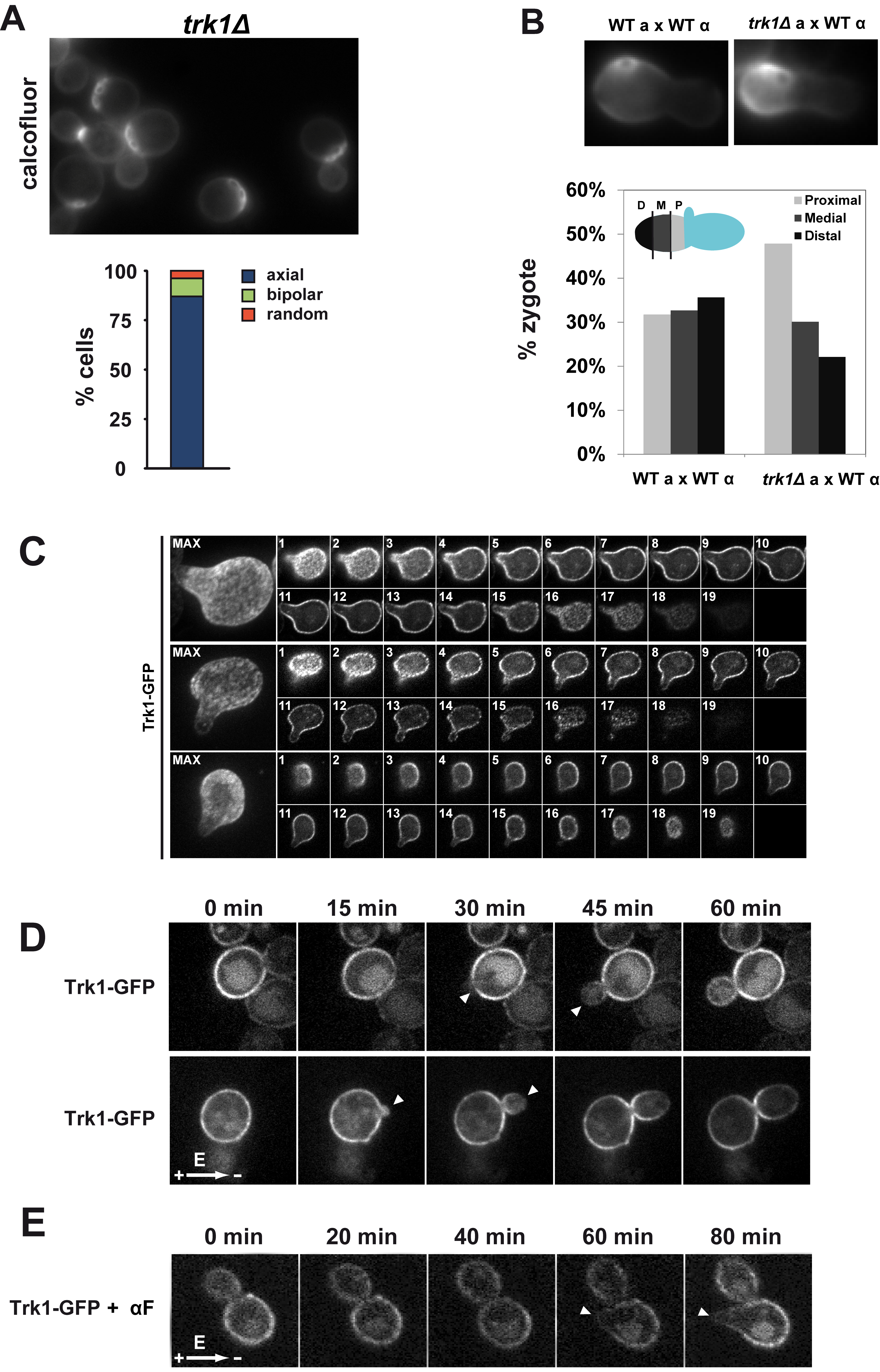

Supplement: Figure S5 — Effects of Trk1 on budding patterns and chemotropism during mating and dynamic localization of Trk1-GFP during bud and shmoo emergence. (A) Axial budding pattern in trk1Δ cells expressing a WT BUD4 (strain AC 134). (B) Position of zygote fusion sites compared to previous bud scars in WT and trk1Δ cells. (C) Trk1-GFP signal is reduced at shmoo tips. Optical sections spaced by 200 nm were used for maximum intensity projections (left, “MAX”). The 19 individual sections are shown on the right. Three representative individual cells are depicted. (D) Changes of localization of Trk1-GFP during bud emergence in presence (in rsr1Δ background) or absence (WT background) of an EF. (E) Changes of localization of Trk1-GFP in shmooing cells exposed to an EF. Note the disappearance of Trk1-GFP at the shmoo tip growing to the anode. (TIF) [file pbio.1002029.s005.tif]

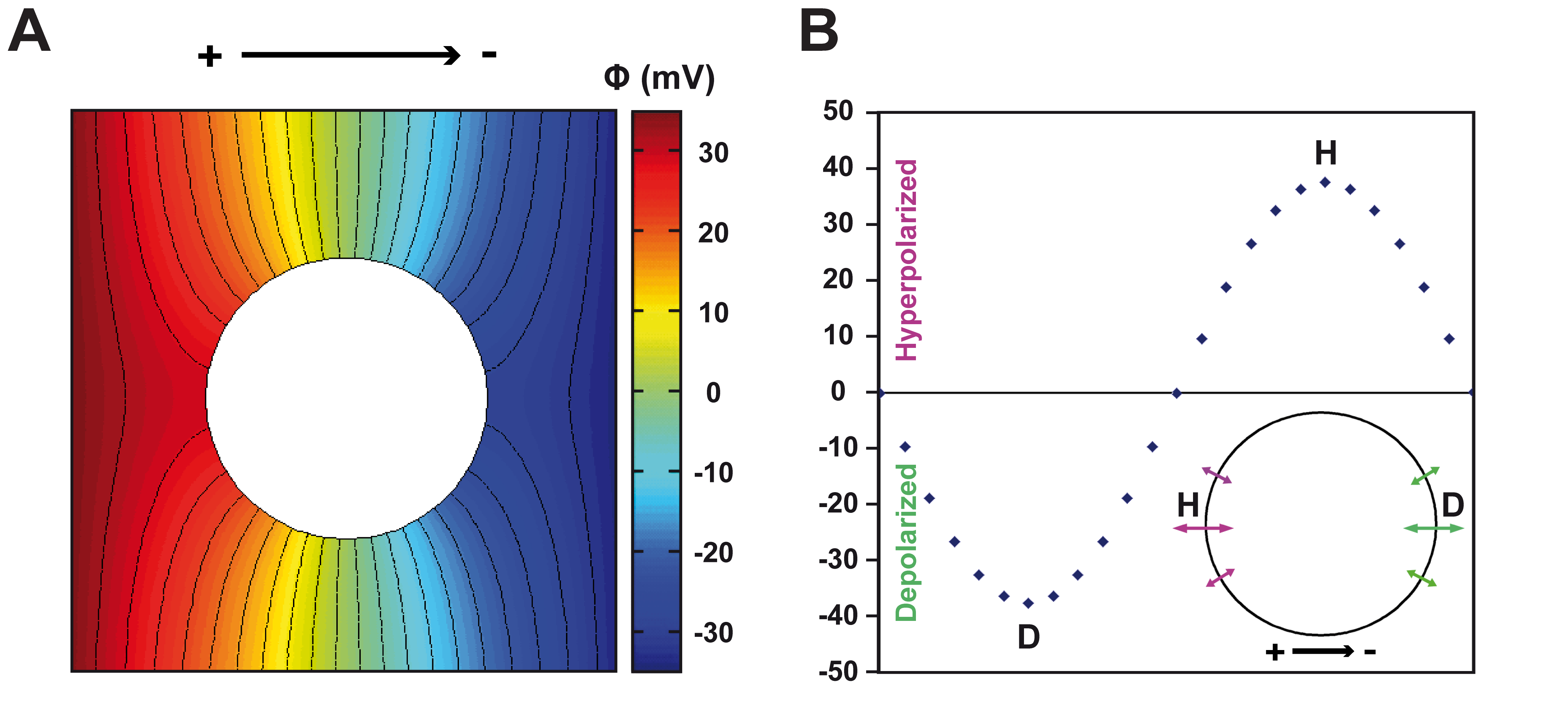

Supplement: Figure S6 — Computational simulation of EF effects on membrane potential predicts a hyperpolarization at the anode-facing side and a depolarization at the cathode-facing side. (A) Computational simulation of the EF-induced electric potential (Φ) landscape around a S. cerevisiae cell created by an EF of 50 V/cm. The cytoplasm is set at an arbitrary homogenous reference potential. The lines represent the equipotentials. (B) Predicted local changes in extra-transmembrane potential created by the EF. (TIF) [file pbio.1002029.s006.tif]

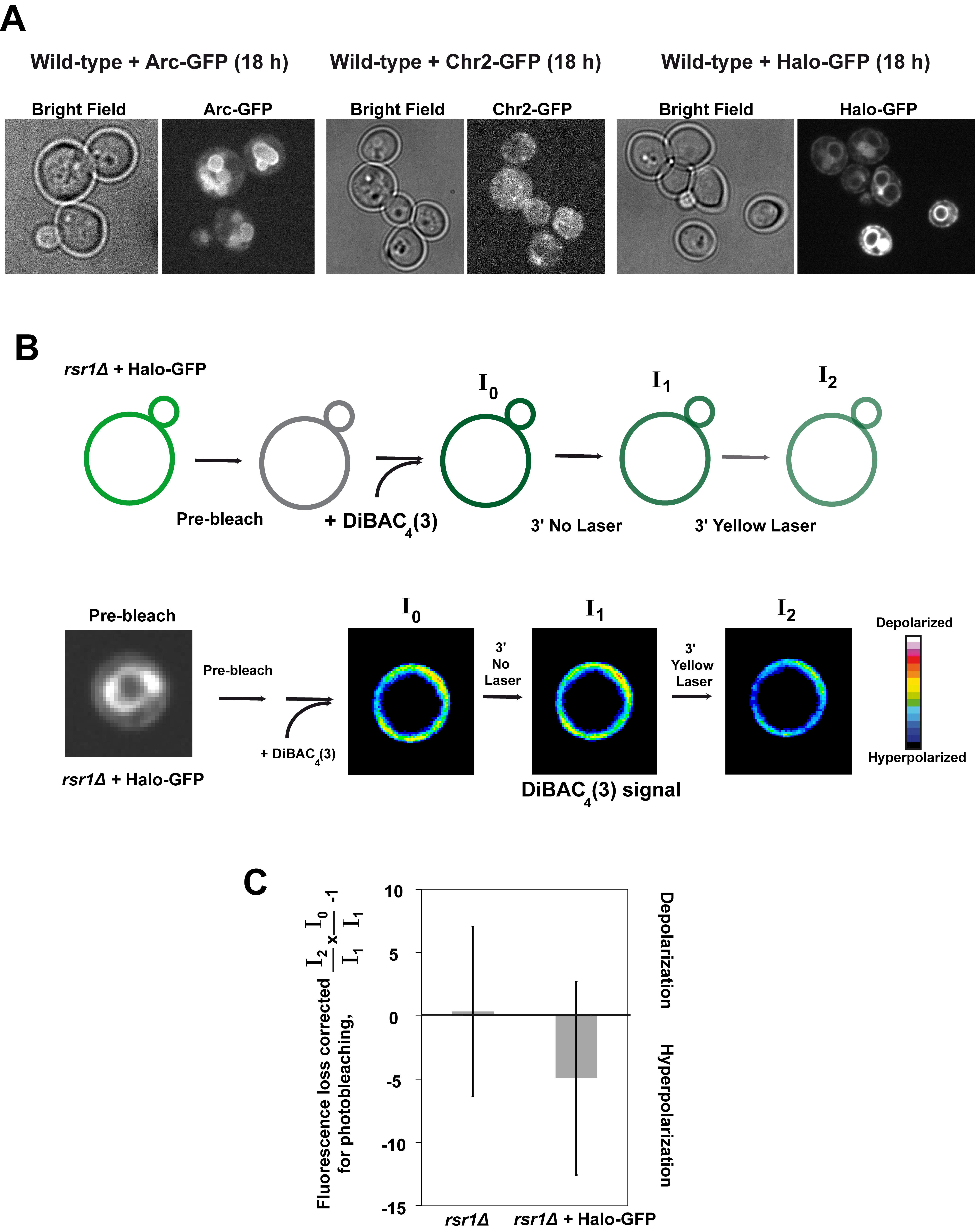

Supplement: Figure S7 — Optimization of optogenetic control of membrane potential in S. cerevisiae. (A) Images of WT cells expressing different opsins (Archaerhodopsin, Channelrhodopsin, and Halorhodopsin—from left to right) tagged with GFP, after 18 h of induction at 25°C. (B) Assay to monitor Halorhodopsin light-induced membrane depolarization in single cells using DiBAC4(3). Cells expressing Halorhodopsin-GFP are placed in a microfluidic flow chamber (see Materials and Methods), and the GFP signal is first bleached with 15 stacks of 5-s exposure with a blue laser. Cells are subsequently rinsed with DiBAC4(3) dye and left to stain for 30 min. Effects of dye photo-bleaching are accounted for by taking single slices spaced apart by 3 min, and measuring membrane intensity subtracted from background before and after the 3-min interval (I 0 and I 1). Hyperpolarization induced by yellow light activation of Halorhodopsin is then assessed by exposing cells to a yellow laser for 3 min, and measuring fluorescence in the green channel (I 2). Specific loss of fluorescence associated with membrane hyperpolarization is computed as , which accounts for dye photo-bleaching, and is expected to be positive upon membrane depolarization and negative upon membrane hyperpolarization. (C) Halorhodopsin activation triggers hyperpolarization of rsr1Δ cells. Fluorescence changes are computed as described in (B). p-Value is 0.079 as calculated by Student's t test. Error bars represent standard deviations, and n≥32 cells were analyzed. (TIF) [file pbio.1002029.s007.tif]

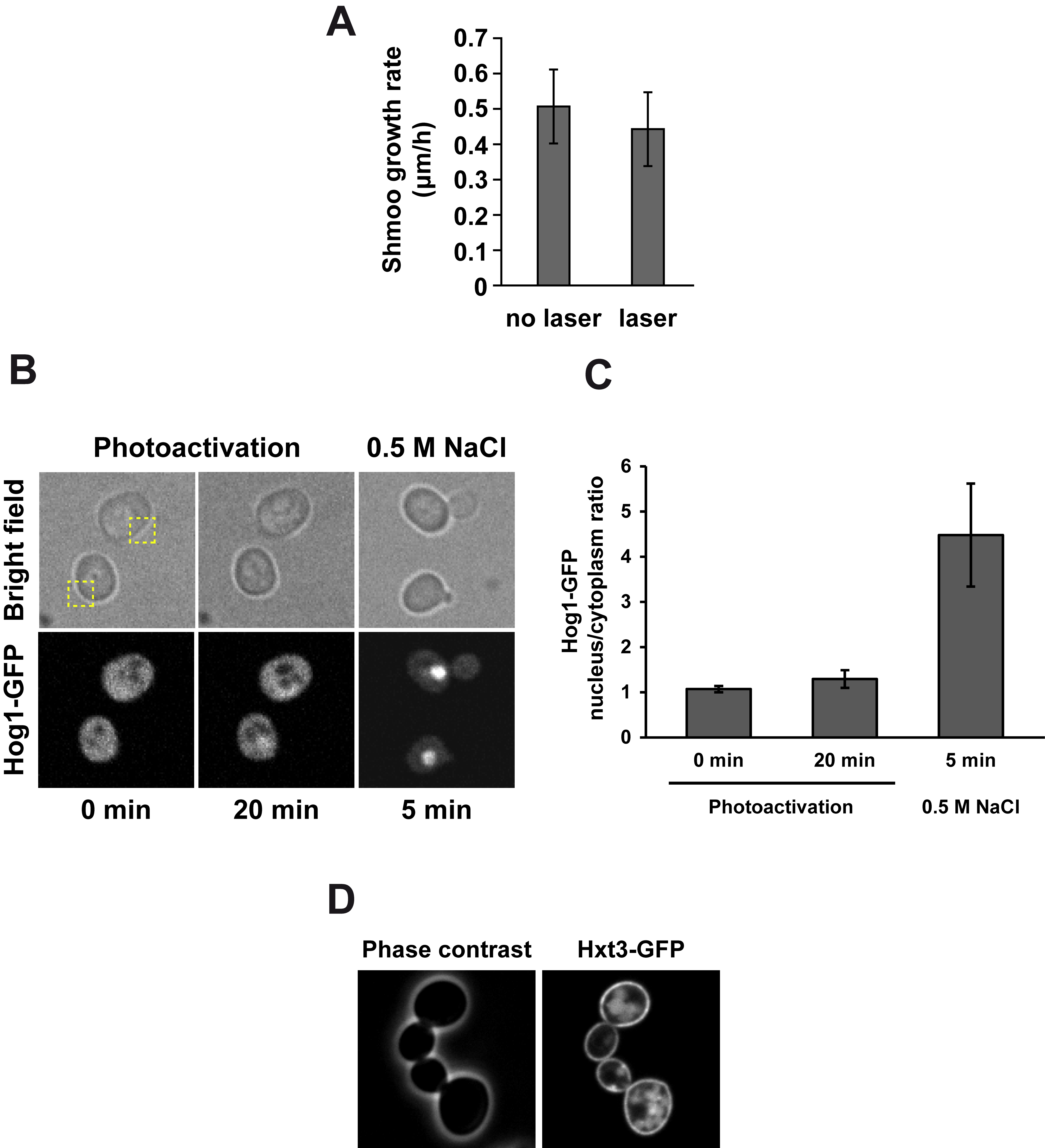

Supplement: Figure S8 — Effects of optogenetic assays on cell physiology. (A) Effect of locally restricted yellow light exposure for 20 min on the growth rate of shmoos. (B) Effect of local yellow light exposure (yellow boxes) for 20 min on stress levels of cells in presence of 50 µM α-factor as measured by Hog1-GFP nuclear accumulation. Osmotic stress (0.5 M NaCl for 5 min) is used as a positive control for stress. (C) Quantification of Hog1-GFP nuclear to cytoplasmic levels. (D) Subcellular localization of Hxt3-GFP expressed under its endogenous promoter in rsr1Δ cells. n>25 cells for each condition. Error bars represent standard deviations. (TIF) [file pbio.1002029.s008.tif]
